# Comparing the brief Holistic Health for HIV (3H+) to the Holistic Health Recovery Program (HHRP+) among people with HIV and opioid use disorder: Results from a randomized, controlled non-inferiority trial

**DOI:** 10.1371/journal.pone.0312165

**Published:** 2024-11-07

**Authors:** Alexei Zelenev, Tania B. Huedo-Medina, Roman Shrestha, Colleen B. Mistler, Frederick L. Altice, Brian Sibilio, Michael M. Copenhaver

**Affiliations:** 1 Department of Internal Medicine, Section of Infectious Diseases, Yale University School of Medicine, New Haven, CT, United States of America; 2 Ikerbasque Research Foundation, Bilbao, Spain; 3 Department of Clinical, Health Psychology and Research Methods, School of Psychology, UPV/EHU, University of the Basque Country, Leioa, Spain; 4 Department of Allied Health Sciences, University of Connecticut, Storrs, CT, United States of America; 5 Institute for Collaboration on Health, Intervention and Policy, University of Connecticut, Storrs, CT, United States of America; 6 Division of Epidemiology of Microbial Diseases, Yale University School of Public Health, New Haven, CT, United States of America; 7 Faculty of Medicine, Centre of Excellence for Research in AIDS (CERiA), University of Malaya, Kuala Lumpur, Malaysia; University of California San Diego School of Medicine, UNITED STATES OF AMERICA

## Abstract

Few evidence-based interventions have been widely adopted in common clinical settings, particularly for opioid-dependent people with HIV (PWH) seeking drug treatment. We developed a brief evidence-based intervention, Holistic Health for HIV (3H+), specifically for ease of implementation and integration within drug treatment settings. In this study, we compared 3H+ to the gold standard, Holistic Health Recovery Program (HHRP+) using a non-inferiority trial. Between 2012 and 2017, 106 participants were randomly assigned to either the brief 3H+ intervention or the gold standard HHRP+. HIV treatment (ART adherence, viral suppression) and risk behaviors (sharing injection equipment, condom use) were compared between the two arms at baseline, end-of-intervention (EOI-12 weeks) and at follow-up (24 weeks). Average treatment effect was calculated based on the difference-in-difference (DID) estimator and a non-parametric bootstrap was used to assess non-inferiority. At the 12-week EOI point, 3H+ was found to be non-inferior to HHRP+ with respect to multiple outcomes: percent sharing syringes and needles (DID:1.4, 95%CI [-18.6,21.5], p<0.01) and attainment of high ART adherence (DID: 9.7, 95%CI: [-13.1, 32.2], p = 0.04). At the 24-week EOI point, 3H+ was found to be non-inferior to HHRP+ with respect to percent sharing syringes and needles (DID: 8.9, [-10.1, 28.30], p = 0.04) and attainment of viral suppression (DID: 18.9, 95% CI:[-7.1, 42.0], p = 0.01). For other indicators, such as consistent condom use, the hypothesis test for non-inferiority was inconclusive at the 12-week EOI (DID: -20.2, 95%CI [-48.9–10.7], p = 0.51). For HIV treatment as prevention to be effective, PWH need to achieve viral suppression. In the absence of this success, they must reduce HIV risk behaviors. The finding that 3H+ was non-inferior to HHRP+ suggests that brief behavioral interventions can be deployed in real world settings to help more efficiently achieve Ending the HIV Epidemic goals.

## Introduction

The volatile opioid epidemic has placed undue strains on public health, including new outbreaks of HIV and HCV related to drug injection. While the prevalence of new HIV cases in the US has not increased, it also has not decreased since 2015 [1] despite wide-ranging efforts. The transition from prescription pain killers to injectable opioids has fueled outbreaks of HIV and HCV, especially where there is suboptimal coverage with medications for opioid use disorder [2–4]. While treatment with medications for opioid use disorder substantially reduces transmission of HIV and HCV [5] and increases treatment engagement along the entire HIV treatment cascade [6,7], people with opioid use disorder (OUD) remain at elevated risk for HIV transmission and suboptimal outcomes due to continued drug- and sex-related risk behavior [8–10].

Settings that provide MOUD are ideal, structured settings to implement secondary HIV-prevention and antiretroviral therapy (ART) adherence interventions among PWH and OUD. To date, however, very few evidence-based interventions have been designed for implementation in this population in the context of outpatient treatment programs, where such interventions are particularly needed. The few EBIs that might be deployed in these clinical settings have not been widely adopted due to numerous barriers (e.g., time constraints, existing treatment routines, personnel demands, patient attributes) that distinguish real-world clinical settings from controlled research settings where interventions are typically tested. Consequently, outpatient treatment programs are rarely able to commit scarce resources toward delivering, monitoring, and evaluating complex evidence-based interventions after they have been developed and tested [11–15]. To guide better adoption and implementation, there has been an evolving need to develop and test briefer, integrated strategies embedded within real world clinical settings [16,17].

Therefore, we have adapted and optimized an evidence-based interventions specifically for implementation among PWH and OUD enrolled in outpatient treatment programs. Through a series of formative studies, we developed the Holistic Health for HIV (3H+) intervention (5 hours of contact) [18], an empirically-adapted, substantially abbreviated version of the Holistic Health Recovery Program for people with HIV (HHRP+: 24 hours of contact) [19], a CDC-recommended evidence based interventions targeting PWH and OUD [20]. The purpose of the present study was to compare the adapted brief 3H+ intervention with HHRP+ [19], a gold standard for secondary HIV prevention for PWH and OUD, using a non-inferiority RCT design [21–23]. We selected a non-inferiority trial design to determine whether replacing an existing evidence-based interventions (i.e., HHRP+) with a significantly abbreviated—yet potentially equally efficacious and likely more cost-effective intervention (i.e., 3H+)–could promote much wider implementation in clinical settings. This design allowed us to test the primary hypothesis that the experimental intervention (3H+) is at least as efficacious (i.e., non-inferior) to the accepted gold standard (HHRP+) [18,19].

## Materials and methods

### Study design

**Fig 1:**
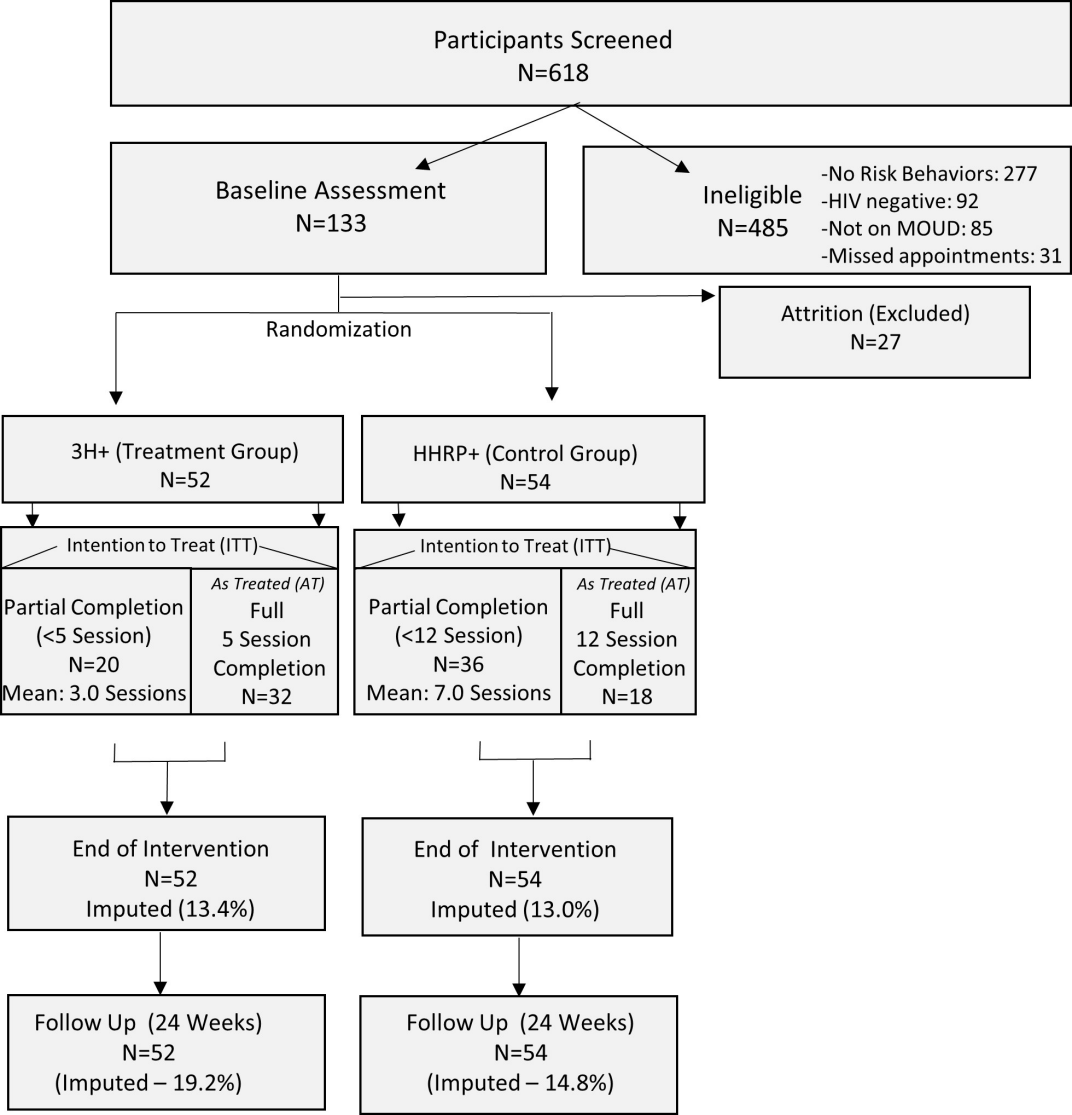
Participant disposition. Medication for Opioid use disorder; 3H+: Holistic Health for HIV; HHRP+: Holistic Health Recovery Program.

The details of this trial have been described in detail elsewhere [24]. A non-inferiority prospective trial was used to compare the efficacy of the adapted 3H+ intervention [18] to the original HHRP+ intervention (Fig 1) [19].

Study participants were PWH with OUD and enrolled in a large community-based methadone clinic in New Haven, CT, with a census of approximately 8,000 patients. Participants were recruited within the same community-based methadone clinic, program as in the original efficacy trial of the HHRP+ intervention [19]. The criteria for eligibility for the participation in the study included: a) ≥18 years of age; b) opioid-dependent and enrolled in MOUD program; c) confirmation of HIV positive status; d) reported drug- or sex-related HIV risk behavior in the past 6 months; e) ability to read and understand the survey questionnaire and provide consent; f) availability for the duration of the study; and g) absence of suicidal, homicidal, or psychotic behaviors.

**Fig 2:**
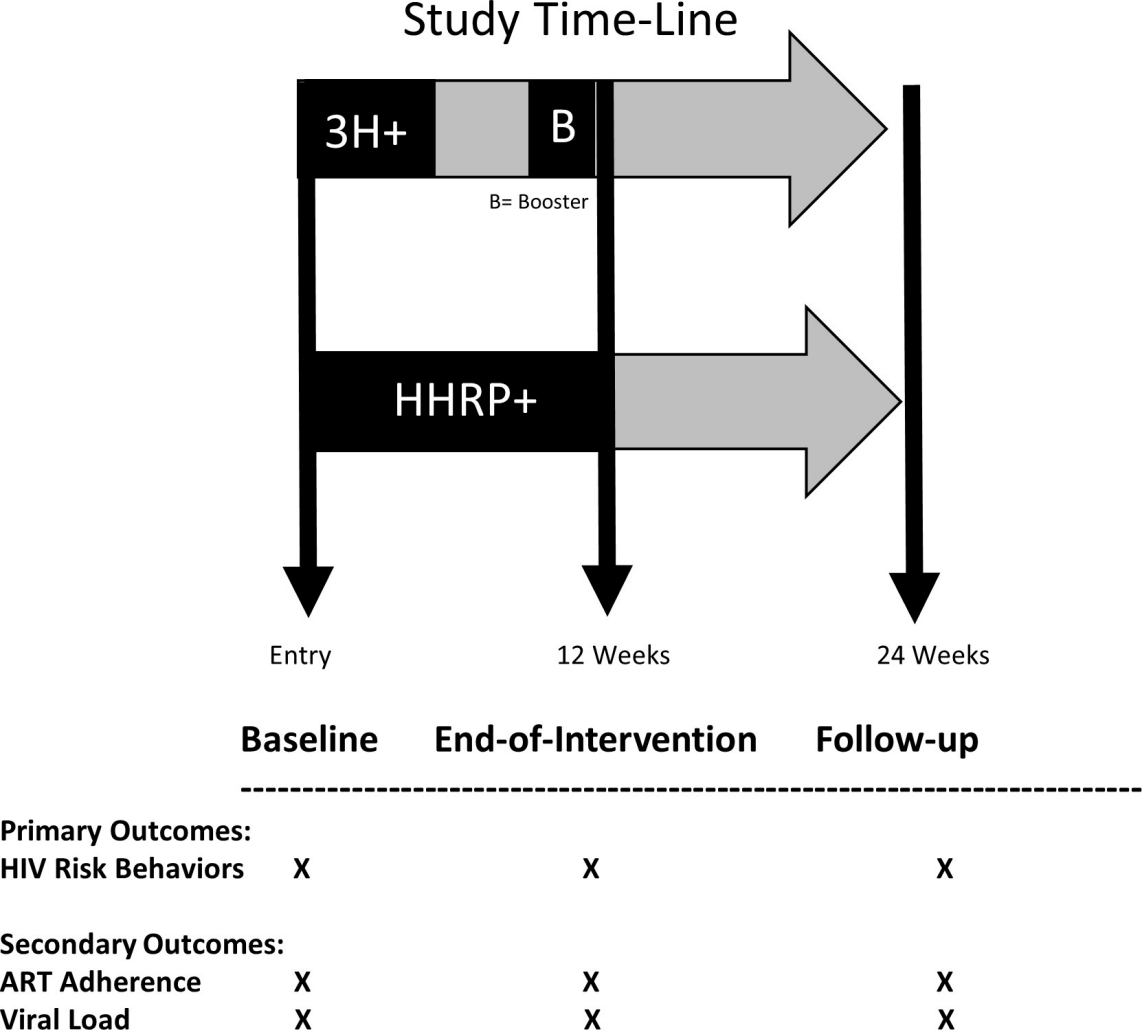
Study design. 3H+: Holistic Health for HIV; HHRP+: Holistic Health Recovery Program for People Living with HIV; B: Booster Session; HIV risk behaviors: Condom Use and Needle Sharing.

Randomization was performed using a computerized “urn” randomization to ensure adequate representation of women and minorities to each intervention arm. The algorithm for probabilistic allocation to treatment and control arms was implemented in Microsoft Access [24,25]. Participant demographics were comparable to the original efficacy trial. Potential contamination was minimized through careful schedule management of the two conditions, as well as independent assessments of participants. The primary outcome time point was 12-weeks, at the end of the intervention (EOI) point. A secondary measurement point was used to assess the short-term durability of intervention outcomes at 24-weeks (twelve weeks following the intervention) (Fig 2).

The study protocol was approved by the Investigational Review Boards (IRB) at the University of Connecticut and at Yale University, and received board approval from the APT Foundation, Inc. (the clinical site). The data were periodically reviewed for safety and efficacy. The risks involved for patients participating in the study were minimal. The clinical trial registration was completed at ClinicalTrials.gov (NCT01741311). A Certificate of Confidentiality was obtained from the National Institutes of Health. A data and safety monitoring board reviewed the data at least annually, as recommended, to ensure data safety and quality control. The data were de-identified after the completion of data collection and human subjects identifying information was removed prior to data analysis. The study Protocol has been provided in the supplementary materials of this publication.

### Sample size

The sample size was based on using a one-sided significance level of 0.025, power of 80%, and the assumption of equal allocation to each intervention condition. Recruitment for the trial was stopped once the target sample size was met with equal allocation of participants among two intervention conditions (1:1). The non-inferiority margins were calculated for each HIV risk and treatment outcome separately using the lower bound of the 95% confidence interval for the average treatment effect, which we simulated by applying a non-parametric bootstrap to the point estimates reported in the original HHRP+ clinical trial [26]. Non-parametric bootstrap is a powerful statistical technique, particularly for small samples, that involves resampling of observations in order to derive estimates of the population parameters, and has been shown to be robust in the in the presence of non-normality and other distributional assumptions [27]. Following the FDA guidelines, the non-inferiority margin represents the smallest plausible benefit of the control that is closest to the null effect [28,29]. Additional technical details, as well as a summary of the results from the original HHRP+ clinical trial are contained in the S1 Appendix.

### Study conditions

#### Holistic Health Recovery Program (HHRP+): Comparison group

HHRP+, the gold standard secondary HIV prevention intervention [19] is a CDC-defined evidence-based interventions [20]. It is composed of 12, two-hour weekly (24 contact hours) manual-guided group sessions with comprehensive HIV risk reduction content that addresses the medical, emotional, and spiritual needs of drug-involved PWH. Each session is designed to last 2 hours and is co-facilitated by two trained clinicians. Co-facilitators address potential motivational conflicts of PWH by providing them with self-protective as well as altruistic reasons for examining and changing their HIV risk behavior and improving ART adherence. Material is presented using a multi-modal presentation style, behavioral games and role-plays, frequent review of the material, and use of memory books.

#### Holistic Health for HIV (3H+): Experimental group

The 3H+ intervention was developed through an iterative process involving the integration of qualitative research [18], theoretical underpinnings [30,31], and findings from a systematic review of the relevant research literature [32]. Using these findings, we adapted HHRP+ to 3H+ using the Assessment-Decision-Administration-Production-Topical experts-Integration-Training-Testing model of intervention adaptation [33]. We then conducted a pilot study of the 3H+ intervention among PWH with OUD who reported drug- or sex-related HIV risk behaviors and who were prescribed ART. The pilot test established feasibility and provided preliminary evidence of the efficacy of the 3H+ intervention in terms of enhancing both sexual- and drug-related HIV risk reduction and ART adherence outcomes [34].

The pilot study involved four, 60-minute weekly group sessions and one 60-minute booster session at 12 weeks (5 contact hours), which is designed to review and maintain HIV risk reduction and ART adherence skills. This theory-based [30,31], manual-guided intervention is a modified coping skills training approach that is delivered in a group modality by two trained facilitators using a motivational enhancement therapeutic style to address high-risk drug- and sex-related HIV transmission risk behaviors and ART adherence. Importantly, it applies cognitive remediation strategies we developed as intervention delivery techniques, which allows us to directly address the otherwise detrimental impact of cognitive dysfunction on intervention engagement and participation.

The participant disposition is presented in Fig 1. The average number of sessions completed by participants in 3H+ was 4.23 (range: 1–5), while the average number of sessions completed by participants in HHRP+ was 8.68 sessions (range: 1–12). Completion rates differed between the two groups with 75% completing all 5 sessions for 3H+ and 33.3% completing all 12 sessions for HHRP+ participants.

### Study procedures

Following informed consent and randomization, participants were invited to attend either the HHRP+ or 3H+ weekly group sessions, both of which were conducted during routine treatment hours at the outpatient treatment programs. Group sessions for each condition were scheduled on different days and times to minimize contamination across conditions. The fifth booster session of the 3H+ intervention was moved to the 12-week time point to ensure an equivalent end-of-intervention time point.

All participants continued to receive ‘standard of care’ medications for opioid use disorder at the outpatient treatment programs regardless of assigned study condition, including: daily methadone or buprenorphine (adhering to the clinic’s standard dosing policies) and case management (maximum of two hours per month of individual face-to-face sessions with a certified drug treatment counselor).

### Assessments

Both risk behavior and HIV treatment outcomes were assessed at baseline and then at 12 weeks (EOI) and 24 weeks (follow-up–short-term durability assessment; Fig 1). The measures based on self-report were administered using computer-assisted structured interviews [35,36]. All risk behaviors were based on self-reported activity over the past 30 days. Injection frequency was defined based on the number of reported times the individual reported injecting either opiates or amphetamines. In addition, the average number of times the individuals shared injection equipment was defined based on the reported number of times respondents shared injecting equipment with other individuals who injected drugs and was used to calculate the difference between baseline and post intervention assessment points.

The number of sexual partners that respondents reported was used create an indicator of whether a person had sex in the past 30 days based on non-zero partners. Consistent condom use was defined as a binary variable based on the individual reporting the use of condoms during every sexual activity and conditional on having at least one sexual partner at baseline and other assessment points. ART adherence was defined as a dichotomous variable based on the individual reporting >95% adherence to prescribed HIV medication using a visual analogue scale. Viral load data were collected within a six-week window of each assessment point, with viral suppression defined HIV-1 RNA<50 copies/m. Data on participant demographic characteristics were also collected, including clinical variables such as attendance at HIV risk reduction services.

### Statistical analyses

Descriptive and inferential statistical analyses were conducted on the demographic variables to describe the sample and determine possible covariates to control for if there were any significant differences between the two conditions in terms of demographics. Independent t-tests and chi-square tests were calculated to determine if there were statistically significant differences (p < 0.05) between the experimental and comparison conditions.

Among the 106 participants, 15.2% of participants (n = 16) had missing data for at least one primary outcome at the 12-week EOI assessment point (the primary outcome point), and 35.8% of participants (n = 38) had at least one missing outcome at the 24-week follow-up assessment point. The structure of the missing data was not associated with the HIV risk behaviors or the demographic variables at baseline. The missing data were assumed to be missing at random and multiple imputations with chained equations were used to impute the missing data [37].

An average treatment effect was defined on the basis of a pre-post difference in proportions for each of the outcomes in 3H+ and HHRP+ groups. Non-inferiority was established by comparing the difference in the difference of the 3H+ and HHRP+ to the pre-specified margin representing the smallest effect of the comparison group. A non-parametric bootstrap was used to construct the confidence interval around each of the treatment effects (3H+ and HHRP+) as well difference of the difference between 3H+ and HHRP+. Finally, the p-value was determined by evaluating the proportion of the bootstrap samples that result in the difference-in-the difference sample estimates to be more extreme that the non-inferiority margin, signifying the probability that the 3H+ was inferior to the HHRP+ intervention. In addition, because intervention completion differed between the two groups, we performed an As-Treated analysis using a subsample of participants who completed all recommended sessions (Fig 1). The statistical analysis was completed in Stata 14 [38].

## Results

The 106 study participants (Table 1) were racially/ethnically diverse (White: 31.1%, African American, 38.7%, and Latino, 29.2%), with a median age of 50 years, and with slightly over half having completed high school (54.7%). Over half of participants had never been married (53.8%). All 106 participants, stratified by 3H+ vs. HHRP+, were enrolled and stabilized in the medications for opioid use disorder program for at least 24 weeks with no differences in medication dose: 82 (77.4%) were on methadone (mean dose: 78.5 mg vs 81.5 mg), 18 (17.0%) were on buprenorphine (mean dose: 11.1 mg vs 10.2 mg) and 6 (5.7%) were receiving monthly extended-release naltrexone injections. The two conditions did not differ at baseline on any variable except that a larger proportion of African Americans were allocated to 3H+. No adverse events or harms to participants occurred during the duration of the trial as a result of participation.

**Table 1:** Baseline characteristics.

Variable	Total Sample	3H+	HHRP+	*χ*^2^ or	p-value
	N = 106 (%)	n = 52 (%)	n = 54 (%)	t-stat	
*Sex*					
Male	63 (59.4)	30 (47.6)	33 (52.4)	0.12	0.720
Female	43 (40.6)	22 (51.2)	21 (48.1)		
*Race/Ethnicity*					
White	33 (31.1)	11 (21.2)	22 (40.7)	7.87	0.049*
African American or Black	41 (38.7)	26 (50.0)	15 (27.8)		
Hispanic or Latino	31 (29.2)	14 (26.9)	17 (31.5)		
Other	1 (0.9)	1 (1.9)	0 (0)		
*Education*					
Not completed H.S.	48 (45.2)	20 (38.5)	28 (51.9)	1.91	0.166
Completed H.S.	58 (54.7)	32 (61.5)	26 (48.1)		
*Marital Status*					
Married	13 (12.3)	4 (7.7)	9 (16.7)	4.31	0.116
Never married	57 (53.8)	33 (63.5)	24 (44.4)		
Separated/divorced/widowed	36 (34.0)	15 (28.8)	21 (38.9)		
*Annual Income*					
$11,000 or below	93 (87.8)	45 (86.5)	48 (88.9)	0.14	0.712
Above $11,000	13 (12.2)	7 (13.5)	6 (11.1)		
*Employment status*					
Working	2 (1.9)	1(1.9)	1 (1.9)	1.22	0.748
Unemployed due to Disability _	63 (59.4)	31 (59.6)	32 (59.3)		
Unemployed (non-Disability)	37 (34.9)	17 (32.7)	20 (37.1)		
Other	4 (3.7)	3(5.8)	1 (1.9)		
*ART Adherence >95% (Last 30 Days)*	65 (61.3)	32 (61.5)	33 (61.1)	0.14	0.885
*Viral Suppression*	74 (69.8)	37 (71.1)	37 (68.5)	0.88	0.347
Sexually Active (Last 30 Days)	79 (74.5)	42 (80.8)	37 (68.5)	2.17	0.141
*Consistent condom use among sexually active*	4 (3.8)	2 (3.8)	2 (3.7)	0.16	0.872
Injection of Drugs (Last 30 Days)	46 (43.4)	24 (46.2)	22 (40.7)	0.18	0.68
*Shared Needles among Active PWID*	26 (24.5)	13 (25)	13 (24.1)	0.61	0.55
*Average Methadone dose (SD)*	79.1 (32.1)	78.5 (24.3)	81.5 (39.1)	0.47	0.669
*Average Buprenorphine dose (SD)*	12.1 (8.3)	11.1 (5.6)	13.3 (10.2)	0.62	0.613

Overall, study participants were retained at relatively high rates at the end-of-intervention (88.7%) and 24-week follow-up (82.1%) assessments (Table 2). Participants in both conditions reported very similar levels of HIV risk and risk reduction across assessment points. Both 3H+ and HHRP+ demonstrated efficacy over time, particularly in terms of decreased needle sharing. Participants in both groups also reported comparably high levels of adherence to ART and viral suppression. In the intervention arm (3H+), a decrease in the number of sexually active population over time contributed to an increase in consistent condom use between end-of-intervention and 12 week follow up relative to the control arm (HHRP+). Patients in both the 3H+ and HHRP+ arms reported lower ART adherence between the 12 and 24 week follow-ups, while the percent of patients who were virally suppressed had larger declines in the HHRP+ than 3H+ groups in the intention to treat sample (N = 106) between the 12 and 24 weeks of follow-ups.

**Table 2:** HIV treatment and risk behavior outcomes by intervention group over the entire observation period (N = 106).

	3H+ (n = 52)			HHRP+ (n = 54)		
Outcomes	Pre-Intervention	End-of-Intervention	24 week Follow-up	Pre-Intervention	End-of-Intervention	24 week Follow-up
**Risk Behavior Outcomes**						
Percent injected drugs in last 30 days	46.3	24.4	32.7	39.9	15.7	11.8
Average number shared syringes or needles in 30 days	2.22	0.22	0.75	1.51	0.20	0.11
Proportion had sex in last 30 days	80.8	69.2	38.5	68.5	53.60	42.6
Percent using condoms consistently	3.8	38.0	59.6	3.7	50.6	50.8
**HIV Treatment Outcomes**						
Percent High (>95%) ART adherence	61.5	70.0	51.5	61.9	61.5	53.7
Percent viral suppression (VL<50)	71.1	81.2	86.0	68.5	82.6	63

The non-inferiority criterion was met when comparing 3H+ to HHRP+ for multiple outcomes at both assessment time points in the Intent-to-Treat (N = 106) sample using difference-in-difference estimator (DID). At the 12-week EOI point, 3H+ was non-inferior with respect to percent sharing syringes and needles (DID:1.4, 95%CI [-18.6,21.5], p<0.01) and percent attaining high ART adherence (DID: 9.7, 95%CI: [-13.1, 32.2], p = 0.04). For other indicators such as percent injecting drugs, percent consistent condom use, and viral load suppression, the non-inferiority test was inconclusive at the 12-week mark due wide confidence intervals of the estimated difference-in-difference (Table 3). At the 24-week EOI point, 3H+ was non-inferior with respect to percent sharing syringes and needles (DID: 8.9, [-10.1, 28.30], p = 0.04), consistent condom use (DID: -20.2, 95%CI: [-48.9,10.7], p<0.03) and percent attaining viral suppression (DID: 18.9, 95% CI:[-7.1, 42.0], p = 0.01) as presented in Table 4.

**Table 3:** Intervention differences over time for risk behavior and HIV treatment outcomes for 106 participants (intention to treat analysis, N = 106).

Baseline vs End-of-Intervention	3H+ (N = 52)		HHRP+ (N = 54)		Difference-in-Difference		Non-Inferiority Margin (Δ)	P-value
Risk Behavior Outcomes	Pre-Post Difference	95% CI	Pre-Post Difference	95% CI	ATE	95% CI		
% Injected drugs Past 30 days	-21.1	(-36.5, -5.8)	-23.6	(-35.1, -11.1)	1.1	(-19.0, 21.7)	9.0	0.219
% Shared syringes or needles	-13.4	(-28.5, 1.9)	-14.8	(-28.0, -1.9)	1.4	(-18.6, 21.5)	26.6	0.008
% Consistent Condom Use Past 30 days	25.8	(11.1,42.4)	46.4	(20.7, 70.8)	-20.2	(-48.9, 10.7)	-20.0	0.511
**HIV Treatment Outcomes**								
%High (>95%) ART adherence	9.6	(-5.8, 23.1)	0	(-16.6, 16.7)	9.7	(-13.1, 32.2)	-11.1	0.041
% Viral suppression (VL<50)	9.6	(-3.8, 23.1)	14.8	(1.9, 27.8)	-5.4	(-24.0, 12.3)	-11.1	0.272

Note: ATE = Average Treatment Effect.

**Table 4:** Intervention differences over time for risk behavior and HIV treatment outcomes for 106 participants (intention to treat analysis, N = 106).

Baseline vs Follow-Up (24w)	3H+ (N = 52)		HHRP+ (N = 54)		Difference-in-Difference		Non-Inferiority Margin (Δ)	P-value
Risk Behavior Outcomes	Pre-Post Difference	95% CI	Pre-Post Difference	95% CI	ATE	95% CI		
% Injected drugs Past 30 days	-13.4	(-30.7, 3.8)	-27.7	(-42.5, -11.1)	12.7	(-10.1, 36.9)	9.0	0.623
% Shared syringes or needles	-9.61	(-25.0, 5.8)	-18.3	(-30.5, -7.3)	8.9	(-10.1, 28.30)	26.6	0.042
% Consistent Condom Use Past 30 days	55.0	(31.3, 77.8)	41.17	(15.0, 66.7)	13.9	(-20.5, 48.2)	-20.0	0.0295
**HIV Treatment Outcomes**								
%High (>95%) ART adherence	-7.69	(-25.0,7.7)	-7.4	(-24.0, 7.4)	-0.36	(-22.9, 20.7)	-11.1	0.181
% Viral suppression (VL<50)	13.46	(0,28.8)	-5.55	(-24.0, 14.8)	18.9	(-7.1, 42.0)	-11.1	0.011

Note: ATE = Average Treatment Effect.

The As-Treated (N = 50) analysis showed a similar pattern of non-inferiority of 3H+ relative to HHRP+ compared with the Intent-to-Treat analysis (N = 106). At the 12-week EOI point, 3H+ was non-inferior with respect to percent sharing syringes and needles (DID: -2.7 95%CI: [-23.3,18.3], p<0.01) and percent attaining high ART adherence (DID 19.8, 95%CI: [7.6, 49.0], p = 0.02), and inconclusive for other indicators in the As-Treated sample (Table 5). At the 24-week follow-up point, 3H+ was non-inferior relative to HHRP+ for percent of sample sharing syringes and needles (DID: 5.2, 95% CI [-16.3, 27.4], p = 0.03), and inconclusive for other indicators, including ART adherence, consistent condom use and viral load suppression in the As-Treated sample (Table 6).

**Table 5:** Intervention differences over time for risk behavior and HIV treatment outcomes for 50 participants (As-Treated analysis, N = 50).

Baseline vs Follow-Up (24w)	3H+ (N = 32)		HHRP+ (N = 18)		Difference-in-Difference		Non-Inferiority Margin (Δ)	P-value
Risk Behavior Outcomes	Pre-Post Difference	95% CI	Pre-Post Difference	95% CI	ATE	95% CI		
% Injected drugs Past 30 days	-34.3	(-54.9, -12.5)	-33.3	(-55.5, -11.1)	-0.8	(-30.2, 30.6)	9.0	0.273
% Shared syringes or needles	-25.6	(-41.4, -10.4)	-22.5	(-37.5, -9.67)	-2.70	(-23.3, 18.3)	26.6	0.004
% Consistent Condom Use Past 30 days	14.3	(0, 33.3)	37.5	(0, 75.0)	-22.2	(-60.4, 14.9)	-20.0	0.540
**HIV Treatment Outcomes**								
%High (>95%) ART adherence	3.1	(-12.5, 21.9)	-16.6	(-38.8, 5.56)	19.8	(-7.6, 49.0)	-11.1	0.021
% Viral suppression (VL<50)	3.1	(-9.4, 18.8)	11.1	(0, 27.8)	-8.0	(-30.2, 10.8)	-11.1	0.412

Note: ATE = Average Treatment Effect.

**Table 6:** Intervention differences over time for risk behavior and HIV treatment outcomes for 50 participants (As-Treated analysis, N = 50).

Baseline vs Follow-Up (24w)	3H+ (N = 32)		HHRP+ (N = 18)		Difference-in-Difference		Non-Inferiority Margin (Δ)	P-value
Risk Behavior Outcomes	Pre-Post Difference	95% CI	Pre-Post Difference	95% CI	ATE	95% CI		
% Injected drugs Past 30 days	-25.0	(-45.8, -3.1)	-38.8	(-66.6, -11.1)	14.6	(-20.1, 48.6)	9.0	0.637
% Shared syringes or needles	-20.5	(-36.9, -4.8)	-25.6	(-41.0, -11.7)	5.2	(-16.3, 27.4)	26.6	0.033
% Consistent Condom Use Past 30 days	41.7	(13.3, 71.4)	27.3	(0, 66.6)	13.9	(-33.3, 57.1)	-20.0	0.071
**HIV Treatment Outcomes**								
%High (>95%) ART adherence	-3.1	(-21.8, 16.5)	11.1	(0, 27.7)	-13.4	(-37.8, 10.06)	-11.1	0.583
% Viral suppression (VL<50)	9.4	(-6.3, 25.0)	11.1	(-10.0, 38.9)	-4.9	(-35.0, 22.6)	-11.1	0.331

Note: ATE = Average Treatment Effect.

## Discussion and conclusion

This study was the first to use a non-inferiority RCT design to assess the relative efficacy of an adapted, brief version of an existing evidence-based intervention (3H+) [18] vs. an original gold standard evidence-based intervention (HHRP+) [19] that targets HIV-infected persons with OUD. This study was designed to provide internal validity by controlling for various confounders and having a target sample powered to detect the difference in outcomes using a similar sample as the original randomized control trial (opioid-dependent individuals enrolled in the same medication for opioid use disorder program) and using several well-validated measures. The abbreviated secondary HIV prevention strategy (i.e., 3H+) was found to be as efficacious as the existing gold standard across assessment points and all outcomes, including needle sharing, condom use, ART adherence, and viral load suppression.

One of the key strategies in the Ending the HIV Epidemic initiative is to control HIV transmission and reduce HIV infection in the US by 90% by 2030. By targeting both injection and sexual risk behaviors, the 3H+ intervention approach aligns closely with the “Ending the HIV Epidemic” strategies focusing on the scale up of treatment as prevention (TasP) and reduction of HIV risk behaviors. The 3H+ intervention is also useful in engaging people with OUD and HIV, which is a crucial for attaining the “Ending the HIV Epidemic”goals. The findings in this study supports efforts to develop efficacious and cost-efficient intervention approaches capable of preventing HIV transmission among high-risk populations such as PWID. The 3H+ intervention requires substantially fewer contact hours per patient (is less costly), and had higher rates of program completion, while meeting the non-inferiority criteria of reducing HIV risk behaviors, as well as achieving the recommended ART adherence and viral suppression levels among HIV-infected patients.

Importantly, prior research has shown disproportionately higher rates of cognitive dysfunction among opioid-dependent persons living with HIV [39–42], which may significantly impede their acquisition and retention of intervention content when using a longer, more complex, behavioral intervention approach. Moreover, other brief interventions have been shown to be as efficacious as longer and more comprehensive interventions [43,44]. In this study, the benefits of the 3H+ outcomes were sustained over the short-term follow-up point, and we did not find any significant differences in the efficacy of the abbreviated intervention vs. the gold standard. Though a markedly smaller proportion of patients in the HHRP+ arm completed all 12 sessions, the As-Treated analysis remained consistent with the non-inferiority criterion. The brief intervention may also promote retention by reducing time demands on medications for opioid use disorder patients, many of whom have to travel to and from the outpatient treatment program. Reducing the demands on clients and incorporating 3H+ sessions into routine counseling sessions may provide an ideal opportunity for expanded reach, particularly since existing drug treatment counselors may be more easily trained to conduct 3H+.

Our findings have substantial implications for the scalability of behavioral HIV prevention interventions in the context of realworld drug clinical settings. Although addiction treatment programs are ideal settings in which to implement HIV prevention programs, the existing evidence-based behavioral interventions have not been widely adopted in such settings due to various organizational barriers (e.g., existing treatment routines, personnel demands, time constraints) and patient attributes (e.g., lack of willingness/ability to participate in lengthy and complex sessions).

Though this study indicates the non-inferiority of 3H+ vs. HHRP+, the study is not without certain limitations. First, the study was conducted at a single site and, thus, may not be generalizable to all outpatient treatment programs. Second, the sample size was too small to examine some factors possibly related to outcomes (e.g., changes in housing status, loss of insurance, etc.), including trends over time in the behavioral outcomes. Third, many of the outcomes were self-reported, though there was no indication that reported outcomes varied as a function of study condition. For the one objective outcome, viral suppression, the direction and the magnitude was similar to the ART adherence outcome, thus reducing this concern. For the As-Treated analysis, the sample size was markedly reduced, especially for the HHRP+ arm, which is not surprising given the relatively greater demands on participants imposed by the HHRP+ intervention. A much larger sample size with a planned As-Treated analysis may have helped disentangle this further. Last, due to some missing data, we selectively used data imputation, which was deemed appropriate when the outcomes met the Missing-Completely-at-Random criteria.

Notwithstanding the noted limitations, this non-inferiority trial supports the use of 3H+ as a much briefer secondary HIV prevention intervention that can be much more readily embedded into common OTPs. Real world implementation, however, is also likely to be based on the relative costs and benefits of such an intervention compared to treatment as usual (i.e., often no HIV prevention intervention). Relatedly, implementation is strongly driven by funding streams that specifically target (1) HIV prevention and (2) addiction treatment among HIV infected persons. Currently, these two funding streams appear only loosely coordinated, and may require a reassessment of how best to respond to the overlapping HIV and OUD epidemics [45]. Moreover, the rapid shifts in behavioral approaches [46–48] and clinical care systems [48–50] during the COVID-19 pandemic demonstrate the growing need to develop and test other intervention modalities (e.g., mHeath) capable of optimally reaching high-risk populations such as HIV-infected PWUD.

The finding that 3H+ was non-inferior to HHRP+ suggests that a more concise behavioral intervention can be deployed in real world settings to help more efficiently achieve Ending the HIV Epidemic goals.

## Supporting information

S1 Checklist (DOCX)

S1 Appendix Calculation of non-inferiority margin. (DOCX)

S1 File (ZIP)

S2 File (PDF)
