# Supplementary material for: Comparing the brief Holistic Health for HIV (3H+) to the Holistic Health Recovery Program (HHRP+) among people with HIV and opioid use disorder: Results from a randomized, controlled non-inferiority trial
Source: PLoS One. 2024 Nov 7;19(11):e0312165. doi: 10.1371/journal.pone.0312165 (PMC11542883; doi:10.1371/journal.pone.0312165)
Supplement: S1 Appendix — (DOCX) [file pone.0312165.s002.docx]

**S1 Appendix**

To estimate the non-Inferiority margin based on the original clinical trial of HHRP+ (Margolin et al., 2003), we simulated a bootstrap confidence interval for the difference between HHRP+ treatment and E-MMP control (Methadone Maintenance Program participation without HHRP+ intervention) based on the frequency count statistics reported in the Margolin et al 2003 trial. However, the frequency count reported in the original study did not adjust for the 30% attrition rate reported by the author in the intervention and control arms, respectively. To account for attrition, we made a conservative assumption that 30% of attrition resulted in non- missing at random (NMAR) mechanism and sampled the missing observations from the baseline distributions in both treatment and control arms based on the frequencies reported at baseline in the original publication. Once we simulated the difference-in-difference in each generated sample of the bootstrap, we calculated the 95% confidence interval based on 10,000 bootstrap samples. The lower bound of the 95% confidence interval was used to define the non-inferiority margin for variables that include consistent condom use and ART adherence, while the upper bound of the 95% confidence interval was used to defined non-inferiority margins for variables that include percent injected drugs and percent shared syringes in our study, representing the smallest effect.

S1 Table: Estimates of the Average Treatment effect and Non-Inferiority Margin.

|  | HHRP+ (N=45) | | E-MMP (N=45) | | Average Treatment Effect | | |
| --- | --- | --- | --- | --- | --- | --- | --- |
| Post Intervention | Pre-Post Difference | 95% | Pre-Post Difference | 95% | Diff-in-Diff | 95% CI | Non-Inferiority Margin ($\Delta$) |
| Percent injected drugs in last 30 days | -47.6 | [-60.0, -35.6] | -37.7 | [-51.1, -24.4] | -10.0 | [-26.7, 9.0] | 9.0 |
| Percent shared syringes or needles | -21.7 | [-40.0, -2.2] | -21.2 | [-40.0, -2.2] | -0.5 | [-26.7, 26.6] | 26.6 |
| Percent using condoms consistently | 24.4 | [6.0, 42.2] | 16.1 | [-4.4, 35.6] | 8.4 | [-20.0, 35.6] | -20.0 |
| Percent with high (>95%) ART adherence | 8.2 | [-13.3, 28.9] | -9.5 | [-28.9, 11.1] | 17.7 | [-11.1, 46.7] | -11.1 |

Source: Calculation based on Margolin et al. (2003)^1^

Appendix References

1. Margolin A, Avants SK, Warburton LA, Hawkins KA, Shi J. A randomized clinical trial of a manual-guided risk reduction intervention for HIV-positive injection drug users. *Health Psychol.* 2003;22(2):223-228.
