# Supplementary material for: Comparing the brief Holistic Health for HIV (3H+) to the Holistic Health Recovery Program (HHRP+) among people with HIV and opioid use disorder: Results from a randomized, controlled non-inferiority trial
Source: PLoS One. 2024 Nov 7;19(11):e0312165. doi: 10.1371/journal.pone.0312165 (PMC11542883; doi:10.1371/journal.pone.0312165)
Supplement: S2 File — (PDF) [file pone.0312165.s004.pdf]

Instructions for Completing the IRB-1 Protocol Application Form

IMPORTANT - Please review the following items before you prepare the protocol application. It will facilitate review of the application form:

- Review the Researcher's Guide section of the IRB website (http://irb.uconn.edu/guide.html) for information and guidance concerning computer/internet based research, consent, deception, recruitment/advertising, etc.

- So that the IRB can properly process your submission quickly and efficiently, please attach the IRB Face Page available at http://www.irb.uconn.edu/forms.html.

- The form now uses the underscore key "___" as a guide for where to provide certain requested information. In cases where the underscore key is used to indicate Yes/No, please erase the underscore and place an "X" next to Yes or No. In cases where the underscore key is used to indicate where to place requested information, please erase the underscore and type in the requested information.

- Read each item carefully and provide the information requested *where* it is requested.

- Place your response BELOW, not within, the box containing each item's description in Sections V, VII and VIII.

- Attach Appendix A to provide a complete list of Key Investigators and Study Personnel.

- DO NOT submit a handwritten application.

- Finally, please DO NOT remove or alter sections of the form that may not be applicable to your study (i.e. do not submit a protocol application with section III deleted if your study is not funded). The document must be provided to the IRB intact. If a section is not applicable to the research, please put "N/A" in that answer section.

- **DELETE this instruction page from the template in the final document.**

Please contact the Office of Research Compliance at 6-8802/0986 with any questions or concerns.

Internal office use only:
Full Board __ Expedited __<br>Protocol #

(IRB-1) Protocol Application for the Involvement of Human Participants in Research

Institutional Review Board, Office of Research Compliance

Whetten Graduate Center, Rm #214, 438 Whitney Road Ext., Unit 1246 Storrs, CT 06269-1246 860-486-8802

SECTION I: General Information

**Nature of Study:** (Place an "X" in the column. Check only one.)	X	Faculty Research		Graduate Research
		Dissertation		Undergraduate Research
		Masters Thesis		Staff Research

Study Title: Secondary HIV Prevention and Adherence Among HIV-infected Drug Users RO1 DA032290

Study Objective (2-3 sentence summary of study:

Using a non-inferiority analytical approach, we will test whether 3H+ (Holistic Health for HIV) is comparable (i.e., within a 10% margin) to the original HHRP+(Holistic Health Recovery Program) in reducing HIV risk behaviors and improving ART(Antiretroviral Therapy) adherence in a randomized controlled comparative effectiveness trial among 256 HIV+ persons in drug treatment who report unsafe injection drug use practices or sexual risk behavior. Using results from specific aim 1, we will conduct a cost-effectiveness analysis of the two interventions in terms of preventing HIV transmission and improving quality of life. Findings will be used to empirically inform the allocation of prevention resources.

PI, Student Investigator, Correspondent Information:

	**Principal Investigator (PI)**	**Student Investigator** (only for Student Initiated Research)	**Correspondent** (primary point of contact for correspondence, if applicable)
Name (First, Last, Degree):			
Department:	CHIP research center		
Mailing Address:	2006 Hillside Drive; Storrs, CT 06269		
Preferred Phone #:			
Emergency Phone # (Required Full Board, More than Min. Risk only):			
Preferred E-Mail Address:			

Very Important: Complete and attach the Appendix A form to list <u>all</u> UConn key personnel engaged in research and other non-UConn investigators.

Section II: Collaborating Institutions/Facilities and Other IRB Reviews

Will the research be conducted <u>only</u> at Storrs and/or the five regional campuses, School of Law, or School of Social Work with no involvement of a collaborating institution? ___ **Yes** _X__ **No (If yes, skip to Section III)**

Collaborating Institutions with a Collaborative Agreement with UConn-Storrs
UConn has formal agreements with the University of Connecticut Health Center (UCHC), Hartford Hospital (HH) and the Connecticut Children's Medical Center (CCMC) that authorize one IRB to take the lead with some research protocols. This decision is made by the IRBs involved, but the PI may request which IRB he/she prefers to be the IRB of record. See the IRB website for additional information. If you are collaborating with one of the institutions listed below, place an X in the appropriate cell to indicate which institution, based on the preponderance of expected enrollment, you are requesting serve as the IRB of record or that independent IRB approval will be sought from each applicable site. If you request that UConn-Storrs be the IRB of record, place an X in the appropriate cell.

Institution Name	% to be enrolled/consented	Requested IRB of Record	Independent IRB Review
UConn Health Center			
Hartford Hospital			
Connecticut Children's Medical Center			
UConn – Storrs		X	

Provide additional comments as needed:

If the PI, Student Researcher or other Key Personnel has an affiliation/appointment with an Institution listed above, please explain: ____

Other Collaborating Institutions/Facilities
If you are collaborating with other sites, provide the name of each institution/facility (e.g. other university, K-12 school, nursing home, tribal affiliation, etc.) and describe the type of involvement of each institution (e.g. recruitment, enrollment/consenting, study procedures, follow-up, data analysis). Indicate if IRB approval/site permission is attached (indicate yes, no, or pending). You will need to obtain IRB approval from every collaborating institution that has an IRB before you can initiate research there.
Note: tabbing out of the bottom right cell will insert another row if needed.

Name of Institution	Describe Involvement	IRB Approval/Site Permission Attached?
Yale University	Collaborating institution (Altice, Co-I; subcontractor)	LOS attached; existing MOA to be updated
APT Foundation	Research Performance Site (methadone program clinic)	LOS attached indicating site permission
Regional Network of Programs	Patient recruitment	

Provide additional comments as needed:

If the PI, Student Researcher or other Key Personnel has an affiliation/appointment with an Institution listed above, please explain: ____

International Research
Will any aspect of the study take place outside of the United States? ___ **Yes** __X_ **No**

(If yes, complete table below)

NOTE: You may need to obtain IRB approval in the country where the research is taking place and/or a Federal-wide Assurance with the Office of Human Research Protections (OHRP). Please see the IRB website for additional information.

**List Location(s)**	**Name of Collaborating Institution/Facility**	**Describe Involvement**	**IRB/Ethics Approval and/or Site Permission Attached?**

Provide additional comments as needed:

If the PI, Student Researcher or other Key Personnel has an affiliation/appointment with an Institution listed above, please explain: ____

SECTION III: Funding

It is the responsibility of the Principal Investigator to notify the IRB via an Amendment (IRB-3) or at Re-Approval, on an IRB-2 form if the funding source changes in any way.

**Funding Source:** (Place an "X" in the column next to the funding source.)				
		Departmental Funds		Human Rights Institute
	**X**	External (including subawards)		Research Incentive Account
		Faculty Grants (Large/Small)		Faculty Start-Up Funds
		Graduate School DDF or EE Award		Investigator Out-of-Pocket
		Office of Undergraduate Research Award		Unfunded

For Internal, UConn Funded Studies:

If the research is supported either in whole or in part by internal funds (Internal Program Support, Office of Undergraduate Research, Research Incentive Accounts, etc) one COMPLETE copy of each grant application (if applicable) must be included with this application.

Name of Internal/UConn Funding Source:	
Principal Investigator**:**	
Grant Title (if applicable and if different from protocol title):	
FRS Account Number (if known and only applicable for Faculty Large and Small Grants funded by Internal Program Support)	
Proposal Number (if applicable, e.g. PD00-0000):	
Grant Status (i.e., pending/awarded):	

Provide any additional comments as needed:

Note: If there is more than one funding source, copy the table format and add the additional funding source.

For Externally Funded Studies:

If the research is supported either in whole or in part by external funds (federal, state or private), one COMPLETE copy of each grant application or contract must be included with this application.

For each funding source, please identify the following:
NOTE: If the PI on the grant/contract is not the PI on this IRB protocol, submit an e-mail with this application in which the PI who is receiving the grant acknowledges use of this protocol under the grant**.**

Name of Funding Source I (if UConn is the recipient of a subaward, list the institution providing the funding then list the primary source of funds):	NIH/NIDA
Principal Investigator of Contract/Grant**:**	Michael Copenhaver, Ph.D.
Contract/Grant Title:<br>(if different from protocol title)	Secondary HIV Prevention and Adherence Among HIV-infected Drug Users<br>RO1 DA032290
FRS Account Number:	FRS# 561507 and
OSP Proposal Number:	OSP# 120174.
Grant/Contract Status:<br>(i.e., pending/awarded)	Awarded by NIH/NIDA 04/01/12

Will funds from this contract/grant be awarded to an individual or institution (via a PSA or subcontract) that will be engaged in human participant research? _x_ **Yes** _ _ **No**
If yes, indicate the name of the institution: ____Yale University; APT Foundation______________________
Provide any additional comments as needed:

Name of Funding Source II(if UConn is the recipient of a subaward, list the institution providing the funding then list the primary source of funds):	
Principal Investigator of Contract/Grant**:**	
Contract/Grant Title:<br>(if different from protocol title)	
FRS Account Number:	
OSP Proposal Number:	
Grant/Contract Status:<br>(i.e., pending/awarded)	

Will funds from this contract/grant be awarded to an individual or institution (via a PSA or subcontract) that will be engaged in human participant research? ___ **Yes** _X__ **No**
If yes, indicate the name of the institution: _____________________________________
Provide any additional comments as needed:

Note: If there are more than two funding sources, copy the table format and add the additional funding source.

SECTION IV: Conflict of Interest (only required for externally funded research)

At the time of proposal submission to the Office for Sponsored Programs (OSP), all investigators and key personnel are required to submit a Significant Financial Interest Review Form to OSP. For more information, please go to the Conflict of Interest Committee website, http://www.compliance.uconn.edu/conflict.cfm.

Is any investigator listed on this protocol required to submit the follow-up form, *"supplemental"* Significant Financial Interest Review Form?   ___ Yes _X__ No

If yes, please identify each individual: ____

SECTION V: Human Participants

Place your responses BELOW, not within, the box containing each item's description.

**How many participants will be enrolled?**<br>If you are enrolling more than one population describe the total enrollment for each. Note: Participants are generally considered to be 'enrolled' when they sign the consent form or have gone through an oral consent process. Therefore, be sure to account for attrition in your enrollment number.

A randomized controlled comparative effectiveness trial among 256 HIV+ persons in drug treatment who report unsafe injection drug use practices or sexual risk behavior

**If applicable, how many potential participants will be screened?**<br>When screening procedures are conducted as part of the consent process, participants that fail to screen will be counted as being enrolled in the study.

All MMPT patients who are interested in the research study will be screened.

**Participant Population(s):**<br>Describe the participant population(s) including gender, ethnicity, age range, income, level of education, and language spoken.

Inclusion/exclusion criteria:

1. At least 18 years of age
2. Opioid-dependent and enrolled in methadone maintenance treatment
3 HIV+ and report unsafe injection drug use practices or sexual risk behavior.
4. Able to read and understand the questionnaires, ACASI (Audio Computer Assisted Self Interview), and consent form
5. Available for the full duration of the study with no anticipated circumstances impeding participation
6. Not actively suicidal, homicidal, or psychotic as assessed by trained research staff under the supervision of a licensed clinical psychologist

Gender. We plan to recruit 60% men and 40% women in order to adequately represent the proportion of men and women in the New Haven community. Recruiting 60% men and 40% women should pose no problem given that the men constitute 61% of the APT Foundation MMP (Methadone Maintenance Program), the patient population from which the subjects for the proposed studies will be recruited. However, given that women typically represent 39% of APT MMP population, we plan to increase our efforts to recruit women into the methadone treatment program by:

(1) posting advertisements at other APT Foundation substance abuse treatment programs (e.g., from the Women in Treatment program);
(2) posting advertisements at primary care clinics serving the community, inner-city health fairs,
and the posting of notices in the community and shelters.

Minorities. We plan to recruit 50% English-speaking minorities; 40% African American and 10% English-speaking Latino/Latina. These target figures are representative of African-Americans and Latino/ Latinas in the community.

**Recruitment:**<br>Describe the recruitment process including *who* will recruit, *when* and *where* recruitment will take place and *how* participants will be identified and recruited (e.g., direct recruitment by study team in person, on the phone, by mail/email/internet, random sampling, referrals from other participants, snowball sampling and/or healthcare providers). Attach copies of all advertisement/recruitment materials for IRB review including phone scripts, web postings, newspaper advertisements. If recruiting at off-campus sites, written permission and/or local IRB approval may be required.

See the above description; we will use an IRB-approved advertisement (TBD as amendment to this application)

Special Population(s):
Identify any special participant population(s) that you will be **specifically targeting** for the study.

Check **all** that apply: (Place an "X" in the column next to the name of the special population.)		Minors		Economically/Educationally Disadvantaged
		Prisoners		Members of the Armed Forces
		Pregnant Women/Neonates		Non-English Speaking
		Decisionally Impaired	**X**	Individuals Living with AIDS/HIV
		UConn Students		Other (Please identify):
		UConn Employees		

UConn Students or Employees:
Are you recruiting students who are in a class you teach or for which you have responsibility? _ **Yes** _X__ **No**
Are you recruiting employees who report to you? ___ **Yes** _X__ **No**

If 'Yes," explain why this population is necessary to the study and indicate precautions taken by the researchers to minimize potential undue influence or coercion:
N/A____

SECTION VI: Drugs/Devices, Genetic Testing, Radiation and Biological Samples

Drug/Device Use
Does the study involve the use of any of the following (check all that apply)?
- An FDA approved drug or medical device ___ **Yes** _X__ **No**
- An investigative/unapproved drug or medical device ___ **Yes** _X__ **No**
- A non-medical device ___ **Yes** _X__ **No**
- A proprietary product ___ **Yes** _X__ **No**
- A biological agent ___ **Yes** _X__ **No**

If yes, please complete the **Drug/Device Supplemental Form** (IRB-1A) and attach it to this application.

Biological Samples
Does the study involve the use of biological samples?
(Either banked or prospectively obtained) __X_ **Yes** ___ **No**
If 'Yes,' you will need to obtain approval from the Biosafety Officer before the study can be initiated. Please attach a copy of the approval letter if approval has already been granted from the BSO.

Genetic Testing
Does the study involve the genetic testing of biological samples? ___ **Yes** __X_ **No**
If yes, please complete the **Genetic Testing Supplemental Form** (IRB-1B) and attach it to this application.

Radiation or Radioisotopes

Does the study involve the use of radiation or radioisotopes? ___ **Yes** __X_ **No**

If yes, you will need to obtain approval from the Radiation Safety Officer before the study can be initiated. Please attach of copy of approval letter if approval has already been granted from the RSO.

SECTION VII: Research Plan

Purpose
State the reason for the study, the research hypothesis, and the goals of the proposed study as related to the research question(s).

The goal of this research study is to conduct a randomized controlled comparative effectiveness trial (RCT) to test the efficacy and cost-effectiveness of an adapted, brief version of an evidence-based intervention (EBI) called Holistic Health for HIV (3H+) vs. the original EBI -- Holistic Health Recovery Program for HIV+s (HHRP+) targeting HIV+ drug users (DUs). The specific aims of the proposed research are outlined below.

Specific Aim 1: Using a non-inferiority analytical approach, we will test whether 3H+ is comparable (i.e., within a 10% margin) to the original HHRP+ in reducing HIV risk behaviors and improving ART adherence in a randomized controlled comparative effectiveness trial among 256 HIV+ persons in drug treatment who report unsafe injection drug use practices or sexual risk behavior.

Specific Aim 2: Using results from specific aim 1, we will conduct a cost-effectiveness analysis of the two interventions in terms of preventing HIV transmission and improving quality of life. Findings will be used to empirically inform the allocation of prevention resources.

Introduction
Provide a clear and succinct summary description of the background information that led to the plan for this project. Provide references as appropriate and, when applicable, previous work in animal and/or human studies. Provide previous UConn protocol number, if applicable.

HIV-infected drug users (DUs) remain a target population as they represent a significant vector for the transmission of new HIV infections (Avants et al., 2004; Margolin et al., 2003), which occur through preventable drug- and sex-related HIV risk behaviors. A number of evidence-based HIV risk reduction interventions are now widely available as complete intervention packages. However, very few evidence-based interventions have been designed for implementation within common drug treatment CBOs, such as methadone maintenance programs (MMPs), where many high-risk HIV-infected drug users seek treatment. Moreover, the few evidence-based interventions that are applicable to drug treatment CBOs are not designed to be “community-friendly” and are therefore unlikely to be implemented as intended or durable within these critical settings. Our team of investigators has developed a significantly shortened version of the comprehensive evidence-based Holistic Health Recovery Program (HHRP; Avants et al., 2004; Margolin et al., 2003). The shortened version, Holistic Health for HIV (3H+) has demonstrated feasibility and acceptability as well as preliminary evidence of effectiveness in an uncontrolled study within a resource-limited drug treatment CBO. Therefore, we now plan to conduct a randomized controlled comparative effectiveness trial (RCT) to test the efficacy and cost-effectiveness of 3H+ vs. the original gold standard EBI -- Holistic Health Recovery Program for HIV+s (HHRP+) targeting HIV+ drug users (DUs).

Design, Procedures, Materials and Methods
Describe the study design, including the sequence and timing of all study procedures. Indicate expected start and completion dates. Include screening procedures, if any. The IRB strongly suggests that investigators incorporate flexibility into the study design to accommodate anticipated events (i.e. explain how missed study appointments can be made up by participants). If the research involves study of existing samples/records, describe how authorization to access samples/records will be obtained. If the study involves use of deception explain the reason why this is necessary. If applicable, describe the use of audiotape and/or videotape and provide justification for use. If this study offers **treatment** for the participants' condition, complete the **Treatment Study Supplemental Form** (IRB-1C) and attach it to this application for review. **If the study includes measures, survey instruments and questionnaires, i**dentify each and, if available, provide references for the measures. Describe what they intend to measure (relate to purpose/hypothesis) and their psychometric properties (e.g., reliability and validity). Identify any that were specifically created for the study.

We will conduct a randomized controlled comparative effectiveness trial (RCT) to test the efficacy and cost-effectiveness of an adapted, brief version of an evidence-based intervention (EBI) called Holistic Health for HIV (3H+) vs. the original EBI -- Holistic Health Recovery Program for HIV+s (HHRP+) targeting HIV+ drug users (DUs). randomized design, balancing for participant gender. We will assess participants at baseline, immediately post-intervention, and at 3-, 6-, and 9-month measurement points following the intervention.
All opioid-dependent patients enrolled in methadone maintenance treatment will be referred for initial screening. As another precaution, we plan to screen 20% more patients than will be needed to meet the proposed sample size. Therefore, 307 patients will be screened (i.e., 5 patients per month). In the screening session, Conducted by a member of the research staff, determination will made as to whether the patient meets study inclusion criteria. If so, the patient will be provided with a description of the study. If the patient agrees to participate, informed consent will be obtained and the patient will be scheduled for the pre-intervention assessment after which the patient will be randomized to one of the two treatment cells. Randomizing participants after they complete the baseline assessment will ensure that recruitment and assessment staff remain blind to the assignment of study conditions. Patients will be randomized using a computerized "urn" randomization to ensure adequate representation of women and minorities. This technique modifies randomization probabilities based on prior composition of intervention conditions and reduces the possibility that imbalances between the intervention conditions on variables such as sex and race will occur by chance.

After providing informed consent, participants will complete a baseline assessment (same for post-test and follow-up assessments) focused on HIV risk behavior. All of the self-report measures given at pre-, post-, and follow-up will be administered using audio computer assisted structured interviews (ACASI). ACASI administration reduces self-report biases (Turner et al., 1996, 1998) and minimizes participant response reactivity (Kalichman & Stevenson, 1997). Importantly, the psychometric properties of ACASI-based assessment are comparable to interviewer assisted assessments among IDUs (Riley et al., 2001). In addition, drug users have been found to show adequate skills to complete ACASI-based assessment (Williams et al., 1998). We have attempted to minimize the response burden of our measures by restricting our instruments to those that are essential to the aims of the proposed pilot trial. These measures are fully described in the grant application. The transcript of the ACASI measures are included in the appendix.

Urine toxicology screens for opiates and cocaine (benzoylecgonine) will also be performed to detect illicit substance use at pre-, post- and at all follow-up assessment points as well as during the intervention. Urine samples will be analyzed using the Abbott Tdx method, which has been shown to be a reliable and precise system. Levels of morphine greater than 200 ng/ml will be regarded as positive for illicit opiates. Levels of benzoylecgonine greater than 300 ng/ml will be regarded as positive for cocaine use. For the range of doses between 13 mg to 130 mg of topically used cocaine, the test will remain positive for at least three days after cocaine use. This is a clinically reasonable sensitivity for illicit cocaine use by either topical (i.e., nasal), freebase, or intravenous routes. Although urine toxicology screens are required standard for all patients enrolled in the methadone program,

these urine toxicology screens will be used for research purposes only and will have no repercussions on a participant's status in the methadone program. However, research staff (under direct supervision of the PI, a licensed clinical psychologist in Connecticut) may take this urine toxicology information into consideration if needed to provide a given participant a referral to a more intensive/appropriate level of clinical care.

Upon meeting eligibility criteria, providing informed consent, verifying comprehension of the terms of the study, and completing the baseline assessment (see Human Subjects section), participants will be randomized to receive, in addition to standard of drug treatment care: (a) 4 weekly group sessions and the 12-week booster session that comprise the 3H+ intervention or (b) 12 weekly group sessions that comprise the HHRP+ intervention. The clinicians supervising the MMP will be blind to which intervention the subject is receiving.

Standard of Drug Treatment Care. Participants in both conditions will receive routine services as part of their enrollment in methadone maintenance, which includes daily methadone and case management consisting of a maximum of two hours of individual face-to-face sessions with a counselor/case manager per month.

Daily methadone. Methadone dose will not be fixed, but instead will adhere to the clinic's standard methadone dosing policies. Methadone dose begins at 25 mg and increases by 5 mg every other day to a target 85 mg, but is adjusted according to individual patient need. Methadone dose will be monitored throughout the study to ensure that it does not vary in any systematic way between the two intervention conditions, and the influence of dose on treatment outcome will be examined in our analysis of the data.

Case management. Case management will consist of a maximum of two hours of individual face-to-face sessions with a counselor/case manager per month. Although FDA regulations permit an even lower level of treatment, we selected this level of treatment for the standard of care condition as this level of counseling closely approximates what is considered standard care in many MMPs, including the APT MMP. As is routine, content of sessions will be based on each counselor's assessment of the patient's psychosocial service needs (e.g., housing, employment, childcare) and will include referral to appropriate social services in the community. Content of the counseling that will also be provided during these face-to-face sessions will be based on topics outlined in a counseling manual developed by Mercer and Woody (Mercer, 1992), and selected on a patient-need basis by the counselor.

Holistic Health for HIV (3H+;Appendix C)**.** Patients assigned to 3H+ will receive the standard of drug treatment care, as described above, plus weekly 60-minute HIV risk reduction groups led by two facilitators trained and supervised by Dr. Copenhaver, the PI and licensed clinical psychologist. Our theory-based (IMB Skills Model of health behavior change), manual-guided, HIV risk reduction and ART adherence intervention is a modified coping skills training approach that is delivered in a group modality by two trained intervention facilitators using a motivational enhancement therapeutic style to address high risk drug- and sex-related HIV risk behaviors and ART adherence. Importantly, it applies cognitive remediation strategies as intervention delivery techniques, which allows us to directly address the otherwise detrimental impact of cognitive impairment on intervention engagement and participation. The 3H+ intervention, which includes four weekly 60-minute group sessions and a 60-minute booster session at 12 weeks, contains only content that relates explicitly to drug- or sex-related HIV risk reduction and ART adherence as outlined below (see 3H+ manual in Appendix C*).* The intervention manual provides facilitators with a counselor toolbox, special instructions, slides and video segments, and materials for experiential practice exercises and demonstrations, quizzes, and stress management. The 3H+ group sessions are (1) Actively Participating in Health Care (focuses on ART adherence), (2) Reducing Risk of IDU, (3) Risk Reduction with Male and Female Condoms, (4)

Negotiating Risk Reduction with Partners, and include a Booster session at 12-weeks which is designed to review and maintain HIV risk reduction and ART adherence skills.

HHRP+ Condition. The comparison intervention condition – the original version of HHRP+– has been identified by the CDC as an EBI (Evidence Based Intervention) and, as such, serves as a 'gold standard' among interventions targeting HIV+ DUs. It is comprised of 12 two-hour weekly manual-guided group sessions with comprehensive HIV risk reduction content that addresses the medical, emotional, and spiritual needs of drug-involved individuals living with HIV. Each session is designed to last 2 hours and is co-facilitated by two trained facilitators. Co-facilitators address potential motivational conflicts of HIV+ individuals by providing them with self-protective as well as altruistic reasons for examining and changing their HIV risk behavior and improving adherence behavior. Material is presented using cognitive remediation strategies55 including a multi-modal presentation of material, behavioral games and role plays, frequent review of material, and use of memory books.

Our research team is keenly aware of the potential for contamination across study conditions. Contamination will be minimized in a couple ways: 1) the two intervention condition sessions will be delivered at different times and different location within the same treatment facility and by the same intervention facilitators; participants in the two conditions will be assessed independently and using ACASI, thus avoiding direct interaction; and 2) couples and housemates will be randomized together. Thus, interactions among participants will not differ from the interactions that would occur under natural circumstances in the treatment program.

In terms of statistical analyses, the primary intervention outcomes will be conducted for: (a) behavioral outcomes (e.g., risk behavior, risk reduction behaviors, illicit substance use) and (b) IMB constructs (i.e., HIV-related Information, Motivation, and Behavioral Skills; Fisher & Fisher, 1992). The framework for testing the study hypotheses will be to compare differences between the two conditions (3H+ vs. Control Condition) on the main outcome variables (e.g., HIV risk reduction behaviors and theoretical constructs) across the 6 assessment points (pre-, post-, 3-, 6-, and 9-month assessment points as well as a weekly check in that consists of 22 questions asking about drug use and sexual behavior over the past week. The questionnaire is administered through ACASI, entering additional theoretically and empirically relevant covariates. The specific statistical procedures we employ will be determined by inspection of the distributions of variables in relation to the assumptions of the statistical tests.

Justification of Sample Size/Data Analysis
Justification of Sample Size: For qualitative and pilot studies, describe how the proposed sample size is appropriate for achieving the anticipated results. For quantitative studies, provide a power analysis that includes effect size, power and level of significance with references for how the sample size was determined. Explain the rate of attrition, with references as appropriate. Data Analysis: For all studies, provide a description of the statistical or qualitative methods used to analyze the data.

Based on our formative research, will expect effect sizes to range from 0.44 to 1.02 (Copenhaver et al., 2006). Therefore, a medium effect size (0.44) was conservatively used to estimate the required sample size for the proposed trial. Further, based on a prior RCT of HHRP+, we assume that the proportion of participants free from HIV risk behavior 24 weeks after the completion of the standard therapy, HHRP+, will be 92%. Given this rate in the 'gold standard' group, we would like to determine if the new intervention, 3H+, is non-inferior to the 'gold standard' group within a non-inferiority margin of 10%. If the null hypothesis is rejected, then we can conclude that the proportion of participants free from HIV risk behavior in the 3H+ group is at least 82%. Using a one-sided significance level of 0.025, power of 80%, and the assumption of equal allocation to each intervention, we will need 116 subjects per arm, or a total sample size of 232, to adequately test our hypothesis. Conservatively allowing for 10% attrition, we will need to recruit 256 total participants. Given that 232 participants will be available for analysis, we will have 81.5% power to test our secondary hypothesis of non-inferiority of 3H+ to HHRP+ when examining high-level ART adherence. For this secondary endpoint, we assume the proportion of participants achieving high-level ART adherence in the HHRP+ group will be 80%. The 3H+ intervention will be considered non-inferior to HHRP+ if the percentage of participants achieving high-level ART adherence in the 3H+ group is not less than a margin of □0=15% compared to the HHRP+ group, or not less than 65%.

List major inclusion and exclusion criteria. Any proposed exclusion criterion based on gender (women of childbearing potential), age, or race must include justification for the exclusion. Describe the conditions under which participants may be removed from the study, i.e., noncompliance with study rules, study termination, etc.

Inclusion/exclusion criteria:

1. At least 18 years of age

2. Opioid-dependent and enrolled in methadone maintenance treatment

3. Report drug- or sex-related HIV risk behavior in previous 6 months

4. Able to read and understand the questionnaires, ACASI, and consent form

5. Available for the full duration of the study with no anticipated circumstances impeding participation

6. Not actively suicidal, homicidal, or psychotic as assessed by trained research staff under the supervision of the PI who is a licensed clinical psychologist in Connecticut.

Risks and Inconveniences
Describe the potential risks to participants (and secondary participants, if applicable) and *steps taken to minimize risks*. Assess the likelihood of the risk occurring and, if it were to occur, the seriousness to the participant. Types of risks to consider include: physical, psychological, social, legal, employment, and financial. Also describe any anticipated inconveniences the participants may experience (time, abstention from food, etc.).

There are minimal risks involved for patients participating in the proposed study and none that are known to be over and above risks of receiving standard methadone maintenance. Any risks involved in completion of rating scales, questionnaires, and collection of urine samples is minimal and adds no risk beyond those normally associated with methadone maintenance treatment. Assessment measures and content of the intervention will involve sensitive and explicit topics related to sexual behavior and substance use. This is necessary given the nature of the intervention and practices that confer risk for HIV infection. However, participants may feel awkward or embarrassed when hearing about or being asked to provide information related to sexual activity. The consent procedure informs participants that sexual behaviors will be explicitly covered in measures and intervention content. Counseling staff will have been trained to observe for signs of subject emotional distress in relation to topic presentation and to employ techniques that prevent, reduce or minimize embarrassment, discomfort, or atypical distress.

Some participants may experience increased anxiety as a result of participating in the intervention. Realistic sensitization and accurate vulnerability self-appraisal are desirable and necessary to motivate behavior change efforts. The investigator is experienced in presenting risk reduction information in ways that link risk to avoidable and modifiable behaviors and risk reduction to the adoption of behavior changes. Counseling staff will observe participants for evidence of becoming overly anxious and will utilize techniques to assure that threat sensitization and vulnerability perceptions are realistic. Research staff will also be knowledgeable of social services that some participants may need, and will assist participants in accessing local resources in areas such as alcohol and other drug abuse treatment, entitlement, and supports for those in or leaving abusive relationships.

Benefits
Describe anticipated benefits to the individual participants. If individual participants may not benefit directly, state so here. Describe anticipated benefits to society (i.e., added knowledge to the field of study) or a specific class of individuals (i.e., athletes or autistic children). <u>Do not include compensation or earned course credits in this section</u>.

The potential benefit in this study is in reduction of HIV risk behavior via the study intervention, which may, in turn, foster improvement in subjects' global functioning. However, subjects may not experience any direct benefits from participation.

Risk/Benefit Analysis
Describe the ratio of risks to benefits. Risks to research participants should be justified by the anticipated benefits to the participants or society. Provide your assessment of anticipated risks to participants and steps taken to minimize these risks, balanced against anticipated benefits to the individual or to society.

The potential benefits appear to far outweigh the potential risks involved in participation. Therefore, the risk/benefit ratio appears to be favorable for this study.

Economic Considerations
Describe any costs to the participants or amount and method of compensation that will be given to them. Describe how you arrived at the amount and the plan for compensation; if it will be prorated, please provide the breakdown. Experimental or extra course credit should be considered an economic consideration and included in this section. Indicate when compensation will be given to participants or when the random drawing will take place.

All subjects will be paid for the time it takes for them to participate in study evaluations as follows: Participants will be reimbursed $25 per week for time spent completing each of twelve weekly brief assessments during the intervention period of participation and $90 each for assessments conducted at baseline, post-intervention, and at 3-, 6-, and 9 month follow-up measurement points. Subjects may be reimbursed a total of $750 for the time we expect it to take for them to participate in all the assessments. All subjects will be paid in cash at the completion of each assessment. . Economic compensation will be identical regardless of the condition to which they are randomly assigned.

Data Safety Monitoring
This is a prospective plan set up by the study investigators to assure that adverse events occurring during studies are identified, evaluated, and communicated to the IRB in a timely manner. Although the investigators initially propose a Data Safety Monitoring Plan (DSMP), the IRB must approve the plan and may require revision of the plan. A DSMP is required for all human studies at the University of Connecticut except for studies determined to be exempt from continuing IRB review. For studies that present more than minimal risk to participants, the IRB will review and determine on a case-by-case basis whether a data safety monitoring board is most appropriate. Please refer to the IRB's policy regarding data safety monitoring *before* completing this section - http://irb.uconn.edu/irb_sop/IRBSOP_submission.html#data_safety_monit.

Issues that should be addressed in the DSMP include the following:
1) frequency of the monitoring
2) who will conduct the monitoring (Under UConn policy a student cannot be the sole person responsible for monitoring the data and safety of the protocol procedures.)
3) what data will be monitored
4) how the data will be evaluated for problems
5) what actions will be taken upon the occurrence of specific events or end points
6) who will communicate to the IRB and how communication will occur

Sample response to issues listed above for minimal risk/slight increase over minimal risk – "Survey results will be monitored by the PI in conjunction with the student investigator once every two weeks (items 1, 2 and 3). Survey responses will be reviewed to monitor for clarity (i.e., the same question is skipped by 5 or more participants). In that case, the question will be revised and an amendment will be submitted to the IRB (items 4, 5 and 6)."

This research will involve testing a behavioral intervention and thus requires a Data and Safety Monitoring Plan (DSMP). To protect human participants in the study from adverse events, and to keep participants informed of any developments in the intervention protocol that may pose unforeseen harm, the PI will collaborate closely with the IRB through all phases of the research. Research staff on-site and the PI will discuss all adverse events in detail. The on-site research staff will immediately report the occurrence of any suspected adverse events to the PI. The PI will inform all members of the research team, and notify the IRB of any adverse events. Should an unforeseen cost or negative consequence of participation be discovered, the IRB will be notified immediately and appropriate changes to the protocol and/or informed consent forms will ensue. All implementation protocols developed for the current research will include a detailed plan for tracking and addressing any unlikely negative impact. Validation and revalidation of the study consent form by the University of Connecticut's IRB is done yearly and involves the PI submitting a written report to the IRB concerning the course of the study including a full description of any adverse events or other unexpected patient-related issues. This report is reviewed by the IRB before the consent form is revalidated. In addition, a

member of the research staff who is independent of the implementation of this protocol and who is hired part-time for this purpose (Marla Genova) will review all data, data collection protocols, data confidentiality procedures, and human participant issues via at least annual meetings convened specifically for this purpose.

Privacy/Confidentiality
Explain how the privacy interests of participants will be maintained during the study (note that privacy pertains to the individual not to the data). Describe procedures for protecting confidentiality of data collected during the study and stored after study closure. Describe how data will be coded. Describe plans for storage and security of electronic data (plan must comply with the University's Policy on the Security Requirements for Protecting University Data at Rest). If identifiable, sensitive information (illegal drug use, criminal activity, etc.) will be collected, state whether a Certificate of Confidentiality will be obtained. Be sure to state whether any limits to confidentiality exist and identify any external agencies (study sponsor, FDA, etc.) that will have access to the data. If participants will be screened, describe the plans for storage or destruction of identifiable data for those that failed the screening.

We will make every effort to insure your privacy and confidentiality. If a participant does NOT choose to participate in this study, all information that he/she has given us will be destroyed immediately. If a participant DOES choose to participate, in all of our study records, he/she will be identified by a number and his/her name will be known only to the researcher. Participants' names will not appear in any publication or be released to anyone without written consent. Participants will be informed in the consent form that they should understand, however, that there is a risk that they will be recognized by other patients or staff involved in the study and that they may be recognized as a participant in a research program. This is no greater than the usual risk of identification that occurs in clinical care at the APT Foundation, Inc. Participants will be informed that each group meeting will be audio-taped and transcribed by a member of the research team in order to ensure that each topic is properly covered by research counselors. Participants will be informed that within one week of each group session, the audio-tape for that session will be destroyed in order to protect your privacy and confidentiality.

The PI has applied for a Certificate of Confidentiality from the National Institute on Drug Abuse (NIDA). This Certificate protects the investigators from being forced to release any research data in which participants are identified, even under a court order or subpoena. Participants are informed that this protection is NOT absolute and that, for example, if the researchers learn about serious harm that could come to a participant or to someone else, we would take steps to protect the person or persons endangered even if it required telling the authorities without permission from a participant. However, we will only disclose information to extent necessary to prevent harm to the person(s) believed to be endangered. When the results of this study are published, participants' names will not be used.

SECTION VIII: Informed Consent

As PI, you are responsible for taking reasonable steps to assure that the participants in this study are fully informed about and understand the study. Even if you are not targeting participants from "Special Populations" as listed on page 4, such populations may be included in recruitment efforts. Please keep this in mind as you design the Consent Process and provide the information requested in this section.

Consent Setting
Describe the consent process including *who* will obtain consent, *where* and *when* will it be obtained, and *how* much time participants will have to make a decision. Describe how the privacy of the participants will be maintained throughout the consent process. State whether an assessment of consent materials will be conducted to assure that participants

understand the information (may be warranted in studies with complicated study procedures, those that require extensive time commitments or those that expose participants to greater than minimal risk).

Participants will all be opioid-dependent patients enrolled in methadone maintenance treatment, and will be referred for possible participation in the study by their counselors, or they may contact research staff directly to indicate their interest. During screening, all subjects will receive an explanation of the study, including its rationale, assessment requirements, and schedule of sessions. A trained member of the research staff who has successfully completed appropriate training in the human participant protection education, as verified by the PI, will explain the study, answer any questions participants may have, review the Consent Form, and obtain signed consent. This interaction will occur individually with each participant in a private office setting at the research site. Participants will be informed that they may take as long as necessary to decide about participation.

Before initiating the intervention phase of the proposed study, the PI obtained a Certificate of Confidentiality from NIDA. The purpose, potential benefits, and potential limitations of this certificate are explained in the text of the Informed Consent form (see appendix).

Capacity to Consent
Describe how the capacity to consent will be assessed for participants with limited decision-making capacity, language barriers or hearing difficulty. If a participant is incapable of providing consent, you will need to obtain consent from the participant's legal guardian (please see the IRB website for additional information).

N/A

Parent/Guardian Permission and Assent
If enrolling children, state how many parents/guardians will provide permission, whether the child's assent will be obtained and if assent will be written or oral. Provide a copy of the script to be used if oral assent will be obtained.

N/A

Documentation of Consent
Specify the forms that will be used for each participant population, i.e., adult consent form, surrogate consent form, child assent form (written form or oral script) or an information sheet. Copies of all forms should be attached to this application in the same format that they will be given to participants (templates and instructions are available on the IRB website).

See the attached Informed Consent form in the appendix.

Waiver or Alteration of Consent
The IRB may waive or alter the elements of consent in some minimal risks studies. If you plan to request either a **waiver of consent** (i.e., participants will not be asked to give consent), an **alteration of consent** (e.g., deception) or a **waiver of signed consent** (i.e., participants will give consent after reading an information sheet), please answer the following questions using specific information from the study:

Waiver (i.e. participants will not be asked to give consent) or alteration of consent (e.g. use of deception in research):

- Why is the study considered to be minimal risk?

  N/A

- How will the waiver affect the participants' rights and welfare? The IRB must find that participants' rights are not adversely affected. For example, participants may choose not to answer any

questions they do not want to answer and they may stop their participation in the research at any time.

N/A

- Why would the research be impracticable without the waiver? For studies that involve deception, explain how the research could not be done if participants know the full purpose of the study.

N/A

- How will important information be returned to the participants, if appropriate? For studies that involve deception, indicate that participants will be debriefed and that the researchers will be available in case participants have questions.

N/A

Waiver of signed consent (i.e. participants give consent only after reading an information sheet):

- Why is the study considered to be minimal risk?

N/A

- Does a breach of confidentiality constitute the principal risk to participants? Relate this to the risks associated with a breach of confidentiality and indicate how risks will be minimized because of the waiver of signed consent.

N/A

- Would the signed consent form be the only record linking the participant to the research? Relate this to the procedures to protect privacy/confidentiality.

N/A

- Does the research include any activities that would require signed consent in a non-research setting? For example, in non-research settings, normally there is no requirement for written consent for completion of questionnaires.

N/A

HIPAA Authorization
On the Storrs campus, the following sites are covered entities under the Health Insurance Portability and Accountability Act:

1. Nayden Rehabilitation Clinic (outpatient physical therapy)
2. Speech and Hearing Clinic
3. Emergency Medical Services (EMS, Ambulance)

If research participants are recruited through these entities, it may be necessary to obtain a Waiver of Authorization to allow you to access records for recruitment and an Authorization to use and disclose Protected Health Information (PHI). Contact the Office of Research Compliance at 860-486-8802 for additional information. **Note:** Student Health Services is not covered by HIPAA; however, FERPA regulations apply.

Principal Investigator Certification

I understand the University of Connecticut's policies concerning research involving human participants and I agree:

1. To comply with all IRB policies, decisions, conditions, and requirements;
2. That this study has been designed, to the best of my knowledge, to protect human participants engaged in research in accordance with the standards set by the University of Connecticut, the United States Department of Health and Human Services, the Food and Drug Administration, and any other sponsoring agency;
3. To obtain prior approval from the IRB before amending the research protocol or the approved consent/assent form;
4. To report to the IRB in accordance with IRB policy, any adverse event(s) and/or unanticipated problem(s) involving risks to participants;
5. To submit the Re-Approval/Completion Form as needed;
6. That my participation and the participation of any co-investigators does/do not violate the University of Connecticut policy on Individual Conflicts of Interest in Research;
7. That each individual listed as study personnel in this application has a) completed the required human subjects training, and b) are knowledgeable of the study procedures described in the protocol;
8. That each individual listed as study personnel in this application possesses the necessary training and experience for conducting research activities in the role described for them in this research study.

Furthermore, by signing below, I also attest that I have appropriate facilities and resources for conducting the study.

**Original Signature of Principal Investigator**	**Date**

**Original Signature of Student Investigator (Only for Student-Initiated Research)**	**Date**

**Original Signature of Medical Monitor (Required for all studies that will be monitored by a Physician)**	**Date**

Department Head Certification

This is to certify that I have read the protocol and believe that there is value in asking and answering these research questions using the approach described in this application. To the best of my knowledge, the researcher(s) have the time, facilities, and expertise to conduct this study.

Original Signature of Department Head<br>(Required for ALL studies, unless grant application/contract is attached; see Section III)	Date
